# AF-SSD: An Accurate and Fast Single Shot Detector for High Spatial Remote Sensing Imagery

**DOI:** 10.3390/s20226530

**Published:** 2020-11-15

**Authors:** Ruihong Yin, Wei Zhao, Xudong Fan, Yongfeng Yin

**Affiliations:** 1 School of Electronic and Information Engineering, Beihang University, Beijing 100191, China; yin1996@buaa.edu.cn (R.Y.); zhaowei203@buaa.edu.cn (W.Z.); XudongFAN@buaa.edu.cn (X.F.); 2 School of Reliability and Systems Engineering, Beihang University, Beijing 100191, China

**Keywords:** geospatial object detection, attention module, encoding–decoding module, lightweight network

## Abstract

There are a large number of studies on geospatial object detection. However, many existing methods only focus on either accuracy or speed. Methods with both fast speed and high accuracy are of great importance in some scenes, like search and rescue, and military information acquisition. In remote sensing images, there are some targets that are small and have few textures and low contrast compared with the background, which impose challenges on object detection. In this paper, we propose an accurate and fast single shot detector (AF-SSD) for high spatial remote sensing imagery to solve these problems. Firstly, we design a lightweight backbone to reduce the number of trainable parameters of the network. In this lightweight backbone, we also use some wide and deep convolutional blocks to extract more semantic information and keep the high detection precision. Secondly, a novel encoding–decoding module is employed to detect small targets accurately. With up-sampling and summation operations, the encoding–decoding module can add strong high-level semantic information to low-level features. Thirdly, we design a cascade structure with spatial and channel attention modules for targets with low contrast (named low-contrast targets) and few textures (named few-texture targets). The spatial attention module can extract long-range features for few-texture targets. By weighting each channel of a feature map, the channel attention module can guide the network to concentrate on easily identifiable features for low-contrast and few-texture targets. The experimental results on the NWPU VHR-10 dataset show that our proposed AF-SSD achieves superior detection performance: parameters 5.7 M, mAP 88.7%, and 0.035 s per image on average on an NVIDIA GTX-1080Ti GPU.

## 1. Introduction

Nowadays, benefitting from the development of remote sensing technology, optical remote sensing images with high spatial resolution are obtained conveniently. There are some widely used remote sensing datasets, such as HRSC2016 [1], NWPU VHR-10 [2], and DOTA [3]. Studies on analyzing and understanding remote sensing images have drawn wide attention in the last few years, which can be applied in searching, traffic planning, rescuing, and so on. 

**Figure 1:**
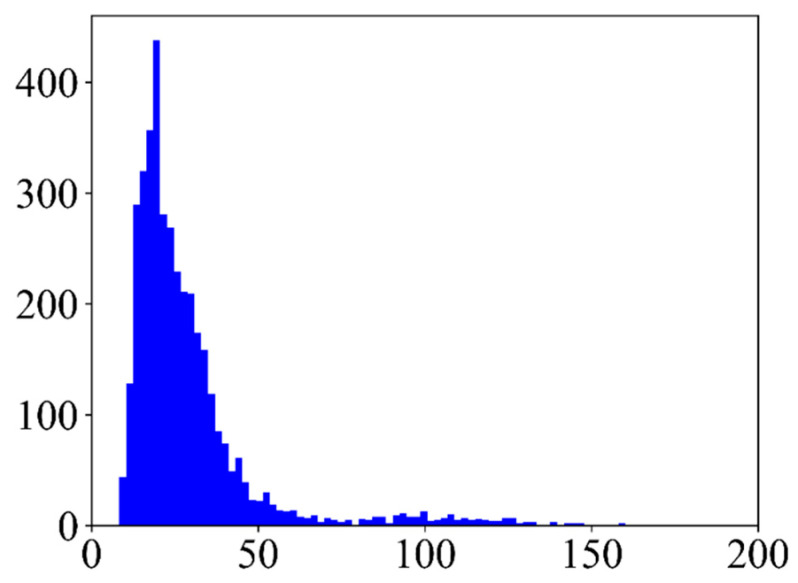
Distribution of target scales in the NWPU VHR-10 dataset. The *x*-axis is the bounding box size of targets defined as $\sqrt{w\times h}$, in which *w* and *h* are the width and height of a target, respectively. The *y*-axis is the number of targets. Images in the NWPU VHR-10 dataset are resized to 300 × 300.

**Figure 2:**
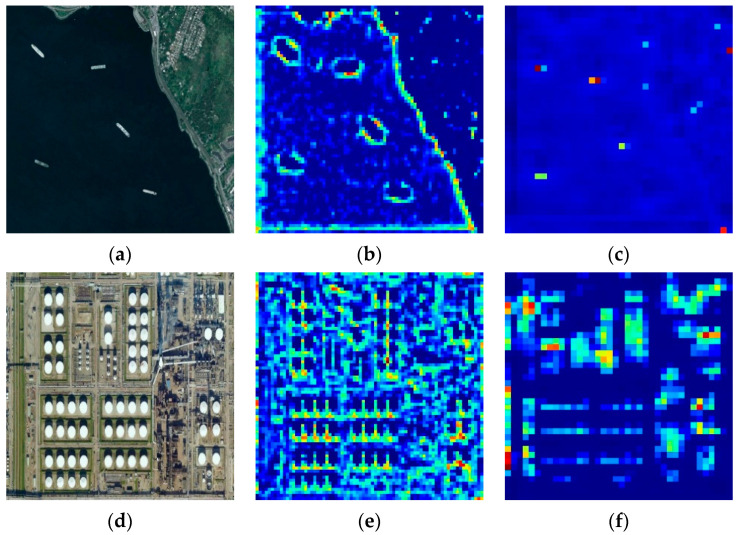
Visualization of feature maps from different convolutional layers. (**a**,**d**) are inputs of the network. (**b**,**e**) show one channel of feature maps with stride 4. (**c**,**f**) show one channel of feature maps with stride 8.

**Figure 3:**
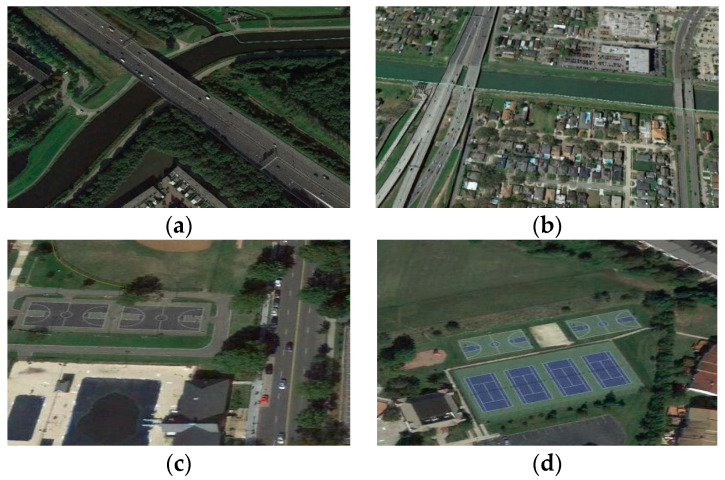
Example images with low-contrast and few-texture targets. (**a**,**b**) are images with bridges. (**c**) is the image with basketball courts. (**d**) is the image with basketball courts and tennis courts.

In recent years, object detection methods based on deep neural networks [4,5,6,7,8,9], especially on convolutional neural networks (CNNs), have made great progress. Mainstream CNN-based object detection methods can be categorized into two classes: two-stage algorithms [5,10,11,12,13,14] and one-stage algorithms [4,6,15,16,17,18]. Two-stage algorithms, like RCNN [11], Fast RCNN [12], Faster RCNN [10], and FPN [5], finish location and classification tasks in two stages. In the first stage, region proposals are generated by selective search [19] or a region proposal network [10] and classified into two classes, i.e., background and foreground. In the second stage, these proposals are refined and classified into a definite category. Due to multi-stage location and classification, two-stage algorithms can get more accurate coordinates for objects. However, these two-stage algorithms take up lots of time and memory. By contrast, one-stage algorithms predict the classification and location of targets in one step, which could achieve real-time detection. The representatives of one-stage methods are the SSD [4], YOLO [6], DSSD [15], and ESSD [16]. With fast speed during inference, one-stage algorithms have been widely used in some scenes that require high efficiency. In some general datasets in which targets are discriminative and large enough, such as PASCAL VOC [20] and COCO [21] datasets, these one-stage methods could reach good performance in both speed and accuracy. Nevertheless, if we apply them to optical remote sensing datasets, the accuracy usually shows a sharp drop. We can summarize the factors in two aspects: (1) because of imaging at a high altitude, there are many small targets in optical remote sensing datasets, and one-stage methods, like the SSD, perform badly for small targets. As shown in Figure 1, if images are resized to 300 × 300, most objects have scales of fewer than 50 pixels. At high-level layers of CNNs, feature maps are down-sampled with a factor of eight or 16 by pooling layers. Therefore, high-level feature maps just have a few features for small targets, which makes it hard to detect them. We visualize the feature maps of intermediate layers in the CNN in Figure 2 to state this: feature maps with stride 4 in the second column have rich features of small targets, and small targets can be recognized easily. However, in the third column, where feature maps have 1/8 resolution of the input, some features of small targets are missing. Hence, detection algorithms have problems recognizing these small targets. (2) Some kinds of targets in optical remote sensing images have low contrast (named low-contrast targets) and lack texture information (named few-texture targets), such as Bridge, Basketball court, and Tennis court (shown in Figure 3). CNNs may have difficulties in extracting features for these targets and detecting them. 

In order to deal with existing difficulties, there are many further works based on one-stage and two-stage methods for geospatial object detection. For example, Zhang et al. [22] came up with a double multi-scale feature pyramid network (DM-FPN) to tackle complex backgrounds in the images and detect small objects, in which a multi-scale region proposal network, multi-scale object detection network, multi-scale training, and inference strategies were all adopted. This structure was complex and the average running time of this method was 3.765 s per image in the DOTA dataset. CACMOD CNN [23] adopted a class-specific anchor and integrated context information for small targets to improve the detection performance in the NWPU VHR-10 dataset. However, it took 2.7 s to deal with one image. Though those methods have improved the detection accuracy, they do not take the detection speed into account and fail to achieve real-time detection. At present, real-time methods with high accuracy are of great significance for disaster prediction, search and rescue, and military information acquisition. There are also some improved methods with fast speed. Zhuang et al. [24] constructed a single shot detection structure with a multi-scale feature fusion module, which achieved a speed of 0.057 s per image in the NWPU VHR-10 dataset. Xie et al. [25] built an optimized one-stage network (NEOON) with feature extraction, feature fusion, feature enhancement, and multi-scale detection strategy at a time expense of 0.059 s per image in the NWPU VHR-10 dataset. However, based on one-stage methods, these frameworks with considerable speed struggle with high accuracy. In short, there is a trend that existing methods only pay attention to speed or accuracy and have difficulty in trading both off. Besides, CNNs have become deeper and deeper, and more complicated. Many CNN-based object detection methods have many parameters and rely heavily on devices with large computational resources. Designing lightweight structures has become an important direction to reduce the memory footprint for mobile and embedded devices, such as space-borne devices, and achieve fast speed. Therefore, in geospatial object detection, the algorithm with a high accuracy, short inference time, and lightweight structure is still necessary. 

**Figure 4:**
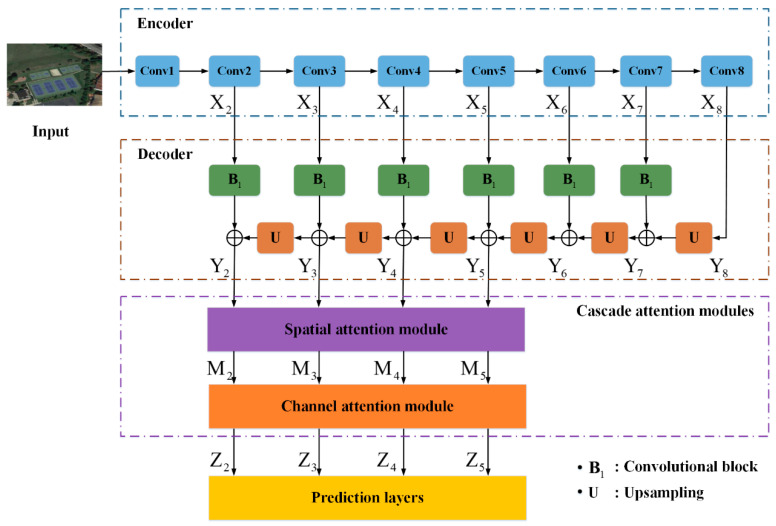
The structure of the accurate and fast single shot detector (AF-SSD).

With our aforementioned description, the main crux of object detection in optical remote sensing datasets is not tackled and there is still a lot of room for improvement. In this paper, we propose an accurate and fast single shot detector (AF-SSD) for high spatial remote sensing imagery, which concentrates on designing a lightweight backbone and extracting effective features for small, few-texture, and low-contrast targets. Taking speed and accuracy into consideration, our AF-SSD is an extension of the one-stage detector, the SSD [4]. The overall structure of the AF-SSD is shown in Figure 4. The main contributions of this paper are as follows:

1. To improve detection accuracy for small targets, a novel encoding–decoding module is proposed. In this module, we fuse low-level features with up-sampled high-level features to gain rich semantic information for small targets. In this way, our method with feature maps at different scales is capable of detecting both small and large targets accurately. Our up-sampling and fusion operations are very light and only add a few parameters to the network. Besides, we only regard some of these feature maps as prediction layers to reduce computation in the following steps.
2. Compared with the background, features of low-contrast targets, like edge information, are not obvious, and they are more likely to be ignored by detectors. For few-texture targets, there is little information on their own and environmental information is of great importance. Therefore, we apply a cascade structure with spatial and channel attention modules to detect low-contrast and few-texture targets precisely. By calculating spatial relationships between pixels and weighting each pixel in a feature map, the spatial attention module can add contextual information for few-texture targets. The channel attention module weights each channel of a feature map by learned weights, which can guide the network to pay attention to important features for few-texture and low-contrast targets.
3. To reduce the number of parameters, we design a lightweight backbone. However, lightweight networks usually have problems in feature extraction and have poor detection performance. To avoid these problems, we also apply some wide and deep convolutional blocks in the backbone to enhance the ability to capture semantic features of the network and keep the detection accuracy constant.

The rest of this paper is organized as follows. In Section 2, our proposed method is introduced in detail. We introduce the details of our experiments, analyze experimental results, and explain the effectiveness of each component in Section 3. In Section 4, we draw conclusions on this work and present future works.

## 2. Materials and Methods

In this section, each part of our AF-SSD in Figure 4 is illustrated in detail. First, we introduce a lightweight backbone in Section 2.1. Then, a novel encoding–decoding module is described in Section 2.2. Next, we illustrate the cascade structure with spatial and channel attention modules in Section 2.3. After that, Section 2.4 presents the prediction layers. Finally, in Section 2.5, loss function during training is explained.

### 2.1. Lightweight Backbone

The original SSD [4] with VGG16 [26] has poor performance, slow detection speed, and a large number of parameters in geospatial object detection, as proved in our experimental results in Section 3. In this part, we will introduce a lightweight backbone to reduce parameters.

**Figure 5:**
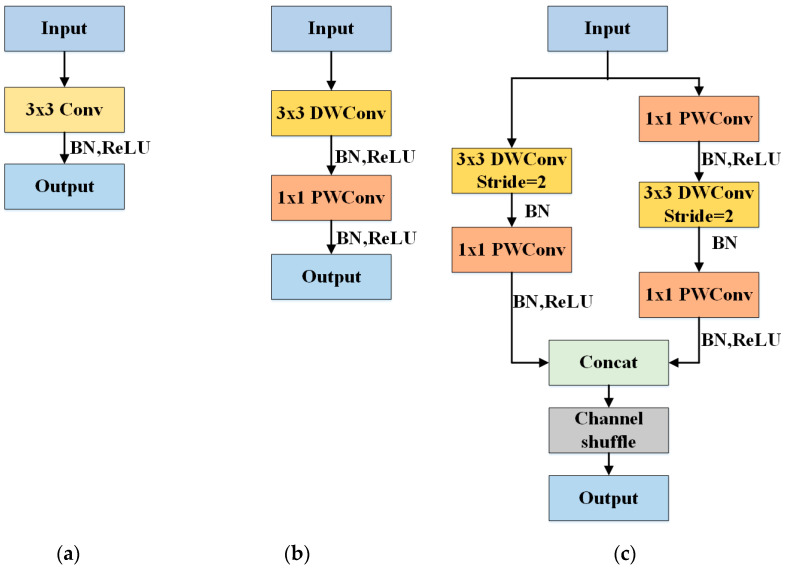
Convolutional blocks. (**a**) Standard convolution with kernel size 3 followed by a Batch Normalization (BN) [27] and Rectified Linear Unit (ReLU). (**b**) Depthwise separable convolution. DWConv: depthwise convolution. PWConv: pointwise convolution. (**c**) A basic unit in ShuffleNetV2.

Our lightweight backbone is shown in Table 1. We apply MobileNetV1 [28] and extra convolutional layers with the basic units in ShuffleNetV2 [29] (shown in Figure 5c) and depthwise separable convolutions (shown in Figure 5b) as the lightweight backbone. In MobileNetV1, standard convolutions (shown in Figure 5a) are replaced by depthwise separable convolutions. A depthwise separable convolution consists of a depthwise and pointwise convolutional layer, which can reduce parameters and deepen the network. Besides, there is a Rectified Linear Unit (ReLU) [30] between depthwise and pointwise convolutional layers. Therefore, depthwise separable convolutions can also add non-linearity to the network. Moreover, the basic unit in ShuffleNetV2 in Figure 5c has multiple branches, which can increase the width of the backbone. Each branch in this unit can focus on different features of a target. Hence, the multi-branch module can gain diversified features of objects. As we know, as the backbone becomes deeper and wider, the semantic information of targets increases. Therefore, after our changes, our light but wide and deep backbone has fewer parameters and can capture rich semantic information for geospatial object detection.

### 2.2. Novel Encoding–Decoding Module

Through the feed-forward computation in the SSD, the resolutions of the high-level layers become smaller and smaller, and some features of small targets are missing. This can be demonstrated in Figure 2: small targets like Ship and Storage tank have apparent features in the feature maps with stride 4 (Figure 2b,e), but some features disappear in the feature maps with stride 8 (Figure 2c,f). Therefore, low-level features with high resolution can retain details for small targets and are specialized for detecting them. However, low-level feature maps just go through a few convolutional layers and have little semantic information, which has a negative influence on the detection performance for small targets. In contrast, as the network goes deeper, high-level feature maps extract rich semantic information of targets. We design an encoding–decoding module in Figure 4 to address this problem. The encoding stage (Encoder in Figure 4) is the feed-forward computation of our lightweight backbone, with the sizes of feature maps decreasing. During the decoding stage (Decoder in Figure 4), to reinforce semantic information for low-level features, our network up-samples high-level feature maps and adds them to low-level feature maps. The decoding stage can be formulated as follows:

(1) $$Y_{i}=\{\begin{array}{cc} B_{1}(X_{i})+U(Y_{i+1}), & 1<i<8\\ X_{i}, & i=8 \end{array}$$

where $B_{1}$ is a standard convolutional block with kernel size 1 to enable $X_{i}$ to have the same channels as $Y_{i+1}$. *U* refers to bilinear interpolation, which is utilized to up-sample feature maps. $X_{i}$ is the output from layer Conv*i* during encoding. $Y_{i}$ is the output after the encoding–decoding module. By combining detailed information in the low-level feature maps with rich semantic information in the high-level feature maps, the encoding–decoding module can increase the high-level information for low-level features and improve the performance for small targets. 

### 2.3. Cascade Structure with Spatial and Channel Attention Modules

#### 2.3.1. Spatial Attention Module

**Figure 6:**
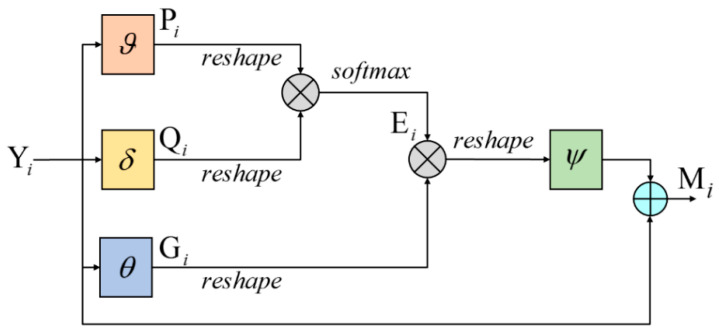
Spatial attention module.

Li et al. [31] and Hu et al. [32] have verified that long-range contextual information can increase information for targets and be beneficial to detect targets. In optical remote sensing datasets, there are some targets with few textures, such as Bridge, Basketball court, and Tennis court. These few-texture targets lack information on their own, so context around them plays an important role in detecting them. In our network, we introduce a spatial attention module (shown in Figure 6) to gain contextual features for few-texture targets. The process is described as follows.

First, the feature map $Y_{i}\in {\mathbb{R}}^{H\times W\times C}$ goes through convolutional layers ϑ, δ, and θ to extract features and reduce channels of the feature map $Y_{i}$. The output feature maps are expressed as $P_{i}\in {\mathbb{R}}^{H\times W\times C_{1}}$, $Q_{i}\in {\mathbb{R}}^{H\times W\times C_{1}}$, and $G_{i}\in {\mathbb{R}}^{H\times W\times C_{1}}$, respectively (*H*, *W*, *C* are the height, width, and channel, respectively, $C>C_{1}$).

(2) $$P_{i}=\vartheta (Y_{i})$$

(3) $$Q_{i}=\delta (Y_{i})$$

(4) $$G_{i}=\theta (Y_{i})$$

Then, $P_{i}$, $Q_{i}$, $G_{i}$ are all reshaped to $N\times C_{1}$, N=H×W. Next, we multiply $P_{i}$ by the transpose of $Q_{i}$ to compute the similarity of these two feature maps. After that, a softmax layer is adopted to transform the values into the interval [0,1] and gain the spatial attention map $E_{i}\in {\mathbb{R}}^{N\times N}$. $E_{i}$ records the correlation between the current position and other positions:

(5) $$e_{ikj}=\frac{\exp(P_{ij}\times Q_{ik}^{T})}{\sum_{j=1}^{N}\exp(P_{ij}\times Q_{ik}^{T})}$$

where $e_{ikj}$ represents the influence that the *j*th position has on the *k*th position in the feature map $E_{i}$. Next, $E_{i}$ is multiplied by the reshaped $G_{i}$, through which each pixel in $G_{i}$ is weighted by pixels in other positions. Therefore, features are enhanced by their contextual information. Then, the feature map is reshaped again to $H\times W\times C_{1}$. In the end, we apply a convolutional layer ψ to transform features and change the number of channels, and adopt a shortcut connection to get the final result $M_{i}\in {\mathbb{R}}^{H\times W\times C}$:

(6) $$M_{i}=\psi (E_{i}\times G_{i})+Y_{i}$$

As explained above, our spatial attention module can calculate the similarity between adjacent positions and weight features in the feature map, which can supplement information for few-texture targets.

#### 2.3.2. Channel Attention Module

**Figure 7:**
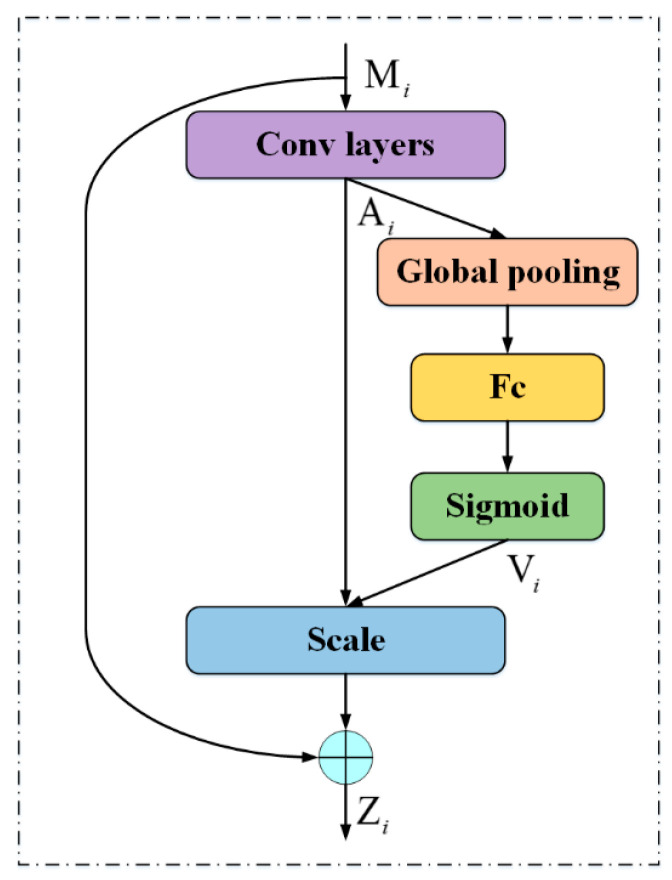
Channel attention module.

Zhang et al. [33] have found that each channel of a feature map represents a sort of feature of targets. These features have different levels of importance in the detection task. As mentioned before, in the remote sensing images, there are lots of targets with few textures and low contrast, and it is hard to detect them. Some of their features are more important than others for detection. In our method, we aim to stress the important features of targets by a channel attention module, which has positive effects on detecting few-texture and low-contrast targets. An overview of the channel attention module is shown in Figure 7. The process is as follows.

The output $M_{i}\in {\mathbb{R}}^{H\times W\times C}$ in the spatial attention module is used as the input in the channel attention module. Firstly, $M_{i}$ passes through a stack of convolutional layers *S* to capture features $A_{i}\in {\mathbb{R}}^{H\times W\times C}$,

(7) $$A_{i}=S(M_{i})$$

Secondly, we generate a channel attention map by exploiting channel-wise relationships of the feature map $A_{i}$. Details of this step are as follows: we first gather information of each channel by the global average pooling g, and then apply fully connected layers *F* to learn relationships between channels. After that, a sigmoid function σ is used to gain the nonlinear relationships between channels, and we gain the channel attention map $V_{i}\in {\mathbb{R}}^{1\times 1\times C}$,

(8) $$V_{i}=\sigma (F(g(A_{i})))$$

Thirdly, we scale each channel with the learned channel attention map. In this stage, we weight the feature map $A_{i}$ with the channel-wise weight $V_{i}$ by element-wise multiplication. We also build a shortcut connection to gain the output $Z_{i}\in {\mathbb{R}}^{H\times W\times C}$ for subsequent classification and location tasks,

(9) $$Z_{i}=V_{i}⊙A_{i}+M_{i}$$

By weighting each channel adaptively, the channel attention module can guide the network to focus on important features and detect few-texture and low-contrast targets precisely.

#### 2.3.3. Cascade Structure

**Figure 8:**
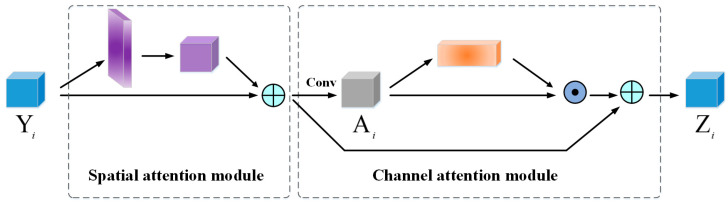
Cascade structure with spatial and channel attention modules.

In Figure 8, we combine both spatial and channel attention modules in a sequential manner, i.e., cascade. The channel attention module treads on the heel of the spatial attention module. Therefore, our cascade structure can not only encode global features, but also emphasize important channels of a feature map to boost the discriminability of features. Experimental results in Section 3 prove the effectiveness of our cascade structure.

### 2.4. Prediction Layers

**Figure 9:**
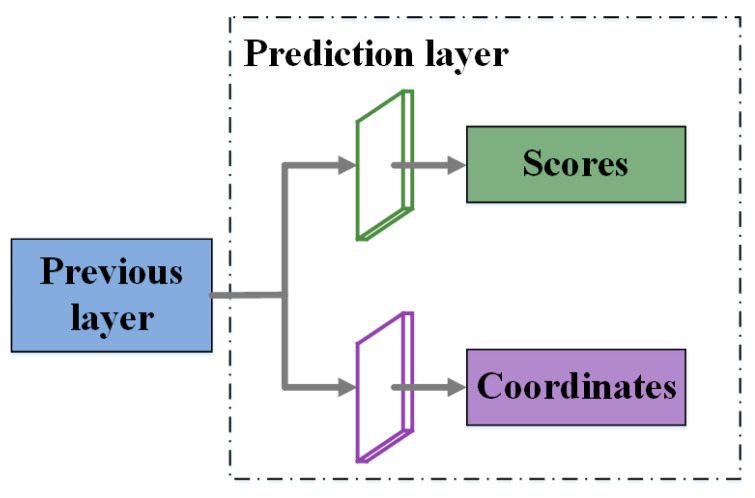
Prediction layer in our AF-SSD: one branch produces scores for the classification task, and another branch outputs coordinates for the location task.

We build four prediction layers on feature maps $\left\{Z_{2},Z_{3},Z_{4},Z_{5}\right\}$, whose strides are {4,8,16,32}, respectively. Low-level feature maps have detailed information for small targets, while high-level feature maps have rich global and semantic information for large targets. In this way, our structure can detect both small and large targets accurately. As shown in Figure 9, each prediction layer has two branches, one for classification and another for location. The classification layer outputs (*K* + 1) category scores $p=(p_{0},p_{1},\ldots ,p_{K})$ for an anchor. The score of each category reflects the possibility that the anchor can be classified into this class. During the inference stage, an anchor is categorized into the category with the highest score. In the location branch, in order to keep translation and scale invariance, the AF-SSD predicts four offsets $t=(t_{x},t_{y},t_{w},t_{h})$ for each anchor $\left(x_{a},y_{a},w_{a},h_{a}\right)$ where $\left(x_{a},y_{a}\right)$ is the center of the anchor, and $\left(w_{a},h_{a}\right)$ is the width and height of the anchor. The predicted box $\left(x_{d},y_{d},w_{d},h_{d}\right)$ is calculated by bounding box regression in Equation (10).

(10) $$\begin{array}{c} x_{d}=w_{a}t_{x}+x_{a}\\ y_{d}=h_{a}t_{y}+y_{a}\\ w_{d}=w_{a}\exp(t_{w})\\ h_{d}=h_{a}\exp(t_{h}) \end{array}$$

For a feature map with height *H*, width *W*, and channel *C*, if the AF-SSD generates *m* anchors with variant scales in each pixel, each prediction layer will yield H×W×m×(K+5) outputs.

### 2.5. Loss Function

During training, we need to determine whether one anchor is matched with a ground truth box. The matched anchors are regarded as positives, while others are negative samples. In our method, we adopt two kinds of matching strategies. First, we match each ground truth box to the anchor with the highest IoU in Equation (11), which can guarantee that each ground truth box has at least a matched anchor.

(11) $$IoU(gt\_box,anchor)=\frac{area(gt\_box)\cap area(anchor)}{area(gt\_box)\cup area(anchor)}$$

where IoU is the intersection over union between a ground truth box *gt_box* and an anchor *anchor*. Second, we match an anchor with a ground truth box if IoU is higher than the threshold (0.5 in our experiments). In this way, a ground truth box may have several matched anchors. Our network can predict classification scores and locations for these matched anchors, which contributes to accurate results. 

We apply a multi-task loss $L_{total}$ on all selected anchors to train the classification task and location task together. $L_{total}$ is calculated as follows,

(12) $$L_{total}=\frac{1}{N_{Pos}}(\sum_{l\in \{anchors\}}\left(L_{cls}(p_{l},u_{l})+[u_{l}\geq 1]L_{loc}(t_{l},t_{l}^{*})\right))$$

(13) $$t^{*}=(t_{x}^{*},t_{y}^{*},t_{w}^{*},t_{h}^{*})$$

(14) $$\begin{array}{c} t_{x}^{*}=\frac{x_{t}-x_{a}}{w_{a}}\\ t_{y}^{*}=\frac{y_{t}-y_{a}}{h_{a}}\\ t_{w}^{*}=\log(\frac{w_{t}}{w_{a}})\\ t_{h}^{*}=\log(\frac{h_{t}}{h_{a}}) \end{array}$$

in which $L_{cls}$, $L_{loc}$ are classification loss and location loss, respectively, and $N_{Pos}$ is the number of selected positive anchors for training. $\left(x_{t},y_{t},w_{t},h_{t}\right)$ is the coordinate of a ground truth box. For an anchor, if the class of the matched ground truth box is $u_{l}$ and the classification score is $p_{l}$, the classification loss $L_{cls}$ is computed as follows,

(15) $$L_{cls}(p_{l},u_{l})=-\log(p_{l})$$

When the anchor is positive, $[u_{l}\geq 1]=1$, otherwise, $[u_{l}\geq 1]=0$. Therefore, the term $\left[u_{l}\geq 1]L_{loc}(t_{l},t_{l}^{*}\right)$ means that only positive anchors are used to calculate the location loss. We use smooth L1 loss as the location loss,

(16) $$L_{loc}(t_{l},t_{l}^{*})=smooth_{L_{1}}(t_{l}-t_{l}^{*})$$

(17) $$smooth_{L_{1}}(x)=\{\begin{array}{ll} 0.5x^{2}, & if\;|x|<1\\ |x|-0.5, & otherwise \end{array}$$

Smooth L1 loss is an extension of L1 loss. For L1 loss, the gradient is constant at 1 or −1. When trainable parameters are close to the optimal, L1 loss still has a large gradient that can result in shaking. Smooth L1 loss tackles this problem by adopting L2 loss [11] near the optimal. Smooth L1 loss is also less sensitive to noise than L2 loss.

## 3. Results and Discussions

We carry out several experiments to estimate the effectiveness of our proposed method. In this part, we compare our method with state-of-the-art methods, like R-P-Faster RCNN [34], NEOON [25], CACMOD CNN [23], and the method in [24]. Besides, we do some ablation studies to verify the effectiveness of each part of our method.

### 3.1. Datasets and Evaluation Metric

#### 3.1.1. Dataset Description

We adopt the NWPU VHR-10 dataset from Northwestern Polytechnical University to verify our proposed method. This dataset consists of 10 categories, i.e., Airplane (PL), Ship (SP), Storage tank (ST), Baseball diamond (BD), Tennis court (TC), Basketball court (BC), Ground track field (GTF), Harbor (HB), Bridge (BR), and Vehicle (VH). It contains 800 very high-resolution remote sensing images: 650 positive images with an object and 150 negative images without any target. We only use 650 positive images for training and inference. Additionally, we split these positive images into three sets, 20% as training dataset, 20% as validation dataset, 60% as testing dataset.

#### 3.1.2. Evaluation Metric

In multi-class object detection, mean average precision (mAP) has been widely used as an evaluation metric. The mAP is the mean of AP values over all categories. It is expressed as follows,

(18) $$precision_{j}=\frac{TP_{j}}{TP_{j}+FP_{j}}$$

(19) $$recall_{j}=\frac{TP_{j}}{TP_{j}+FN_{j}}$$

(20) $$AP_{j}=\int_{0}^{1}precision_{j}(r_{j})d(r_{j})$$

(21) $$mAP=(\frac{1}{K})\sum_{j=1}^{K}AP_{j}$$

If the IoU between a detected box and a ground truth box is over 0.5, the detected box is considered as a true positive, otherwise, it is a false positive. For category *j*, $TP_{j}$, $FP_{j}$, $FN_{j}$ are the number of true positives, false positives, false negatives, respectively. $r_{j}$ is the recall of category *j*. *K* represents the number of categories, K=10, in the NWPU VHR-10 dataset. The detection method with both high precision and high recall is considered an ideal algorithm. However, precision and recall are contradictory. As the recall increases, the precision drops. Therefore, taking both precision and recall into consideration, mAP is used to evaluate our proposed method.

### 3.2. Implementation Details

Our proposed method is implemented with the PyTorch framework. We initialize MobileNetV1 with the pre-trained model in the ImageNet classification dataset [35]. Other convolutional layers are initialized with Kaiming normalization [36]. The batch size is eight in all experiments. We employ stochastic gradient descent (SGD) with momentum 0.9 and weight decay 0.0005 to optimize our network. Specially, we apply a warm-up learning rate strategy. The initial learning rate is 0.0001 for the first 500 iterations. After that, the learning rate is changed to 0.001. When the iteration reaches 30,000, 40,000, and 50,000, we lower the learning rate by 0.1. We train our network for 60,000 iterations in total. 

The input of the AF-SSD is resized to 300 × 300. We only generate anchors on feature maps $\left\{Z_{2},Z_{3},Z_{4},Z_{5}\right\}$. We use small anchors for low-level feature maps, while anchor scales of high-level feature maps are large. The minimum anchor scale $s_{min}$ for each feature map is [15, 30, 60, 111], respectively, and the maximum anchor scale $s_{max}$ is [30, 60, 111, 315], respectively. The aspect ratios of anchors in $\left\{Z_{2},Z_{3},Z_{4},Z_{5}\right\}$ are $\left\{\frac{1}{2},1,2\right\}$, $\left\{\frac{1}{2},1,2\right\}$, $\left\{\frac{1}{3},\frac{1}{2},1,2,3\right\}$, $\left\{\frac{1}{3},\frac{1}{2},1,2,3\right\}$, respectively. The width and height of an anchor are computed as follows,

(22) $$w_{a}=s_{min}\sqrt{a_{r}}$$

(23) $$h_{a}=s_{min}/\sqrt{a_{r}}$$

in which $a_{r}$ is the aspect ratio of an anchor. When the aspect ratio is 1, we add one anchor with the same height and width at $\sqrt{s_{min}\times s_{max}}$. Therefore, there are {4, 4, 6, 6} anchors in each position of $\left\{Z_{2},Z_{3},Z_{4},Z_{5}\right\}$, respectively.

As mentioned before, one-stage algorithms generate many anchors in each feature map. However, during the matching step, only a few anchors are positive samples, and a large number of anchors are negative samples. There is a severe imbalance between positive and negative samples. To cope with this problem, hard negative mining is introduced to choose negative samples, instead of using all negative anchors during training. First, hard negative mining calculates the confidence loss according to classification scores of negative samples. Then, these anchors are sorted in descending order in terms of the loss. In the end, we pick out the top anchors with high losses for training, and the ratio of the positives and negatives is 1:3.

In the inference stage, we adopt non-maximum suppression (NMS) [37] with an IoU threshold of 0.45 to remove redundant boxes. The computing environment is an NVIDIA GTX-1080Ti GPU with 11 GB memory. The code of our method is available at https://github.com/yinlily12/AF-SSD.

### 3.3. Experimental Results and Discussions

**Figure 10:**
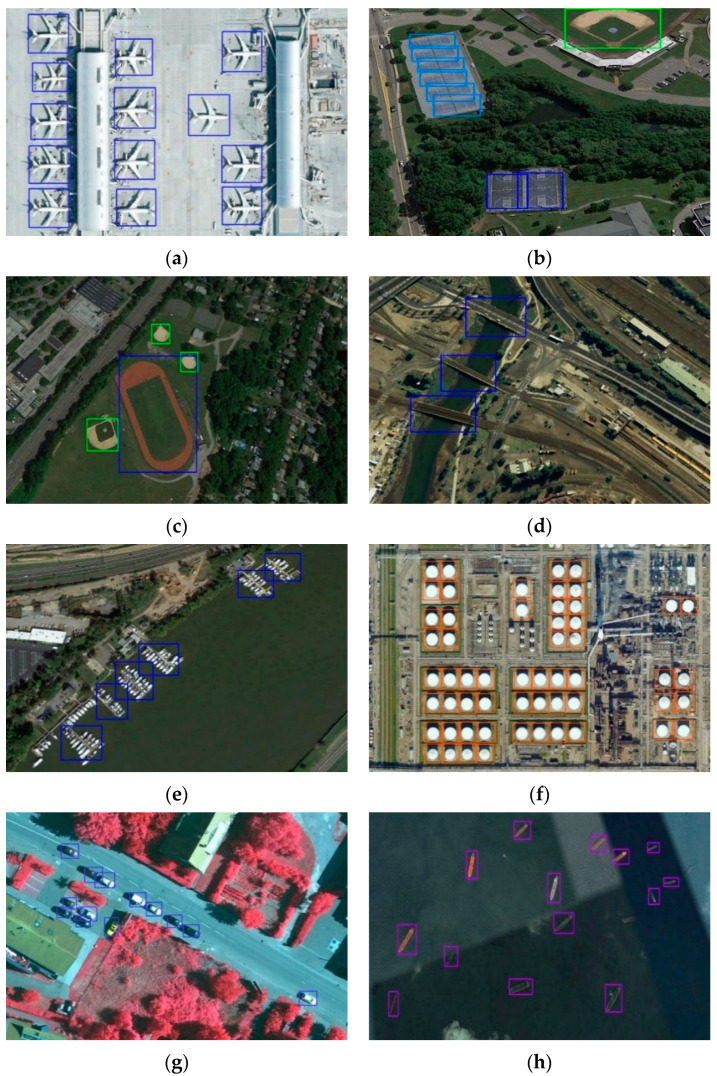
Detection results of the NWPU VHR-10 dataset. (**a**) Airplanes. (**b**) Basketball courts, Baseball diamonds, and Tennis courts. (**c**) Ground track fields and Baseball diamonds. (**d**) Bridges. (**e**) Harbors. (**f**) Storage tanks. (**g**) Vehicles. (**h**) Ships.

We show some detection results of the NWPU VHR-10 dataset in Figure 10. In the complex environment, our proposed method can recognize objects of all classes accurately. As shown in Figure 10a, overexposure targets like Airplanes can be found with precision. Our method makes contributions to the detection of densely distributed objects such as Tennis courts and Storage tanks in Figure 10b,f. Meanwhile, our AF-SSD could recognize small targets well, like Storage tanks, Vehicles, and Ships in Figure 10f–h. The method is also very effective for low-contrast objects like Bridges in Figure 10d, and few-texture targets like Tennis courts and Basketball courts in Figure 10b.

We evaluate the detection performance of our AF-SSD in the NWPU VHR-10 dataset and compare it with state-of-the-art methods in Table 2. Our AF-SSD achieves 88.7% mAP. We bold the two highest values of each column, and the results of our AF-SSD are almost in the top 2. Among the results in Table 2, we draw conclusions as follows,

(1) Two-stage methods like R-P-Faster RCNN, Faster R-CNN, and CACMOD CNN [23] have higher precision than one-stage methods YOLOv2, NEOON, SSD, and the method in [24].
(2) Compared with one-stage methods YOLOv2 [38], NEOON [25], SSD [4], and the method in [24], our method has the highest performance, 28.2%, 11.2%, 8.2%, and 4.9% higher than them, respectively. Particularly, for some categories, like Airplane, Ship, Tennis court, and Bridge, the AP values of our method gain significant improvement and show the superiority of our framework.
(3) The AF-SSD also outperforms the two-stage methods R-P-Faster RCNN [34] and Faster R-CNN [10] by 12.2% and 7.8%, respectively, and has better performance in the categories Airplane, Ship, Storage tank, Tennis court, Ground track field, Harbor, Bridge, and Vehicle. This verifies that our method has outstanding performance for small, low-contrast, and few-texture objects.
(4) Besides, the mAP of the AF-SSD is 34.1% higher than the mAP of COPD [2] and 16.1% higher than the mAP of RICNN [39], and the AP values over all categories of the AF-SSD surpass the AP values of these two methods.

Hence, the quantitative results in Table 2 prove that our method is very effective, which can recognize each kind of object accurately in the optical remote sensing dataset. 

The average running time of these geospatial object detection methods is summarized in Table 3. Our AF-SSD reaches 0.035 s per image, which outperforms the two-stage methods R-P-Faster RCNN (0.150 s) and Faster R-CNN (0.430 s) significantly. In particular, our structure is nearly 80 times (0.035 s vs. 2.700 s) faster than the two-stage method CACMOD CNN, though CACMOD CNN has a better mAP. Our AF-SSD also has a shorter average running time than some one-stage methods, such as the SSD, NEOON, and the method in [24]. In contrast to YOLOv2, the AF-SSD takes more time during inference, but it is still worthwhile because, considering the tradeoff between accuracy and speed, our AF-SSD achieves a 28.2% improvement in mAP with a relatively fast speed. Compared with two-stage methods, like R-P-Faster RCNN, Faster R-CNN, and CACMOD CNN, one-stage methods (like YOLOv2 and NEOON) have a faster speed but lower accuracy, which matches the conclusion that we introduced in Section 1.

In conclusion, compared with state-of-the-art methods, our AF-SSD can reach great accuracy with high computational efficiency in geospatial object detection, which can be used to detect targets in real time.

### 3.4. Ablation Study

We do a series of ablation studies to explain the effectiveness of each part in our proposed structure, including the lightweight backbone, the novel encoding–decoding module, the cascade structure with spatial and channel attention modules, and the contribution of Leaky ReLU [40]. The experiments for comparison are set as follows,

1. Light backbone: we apply the lightweight backbone in Section 2.1 to illustrate its effectiveness. There is no encoding–decoding module or cascade attention module in this model.
2. Light backbone + EDM: an encoding–decoding module (EDM) is added to Setting 1 to enhance low-level features. Due to a large number of small targets in remote sensing images, we only apply feature maps $\left\{Z_{2},Z_{3},Z_{4},Z_{5}\right\}$ to prediction.
3. Light backbone + EDM + Cascade: we apply a cascade structure with spatial and channel attention modules before prediction layers on Setting 2, which is designed to gain global and important features for both low-contrast and few-texture objects.
4. Light backbone + EDM + Cascade + Leaky ReLU: we replace ReLU in the network by Leaky ReLU to alleviate the negative influence that ReLU has in the interval (−∞,0). This structure is our AF-SSD.

**Figure 11:**
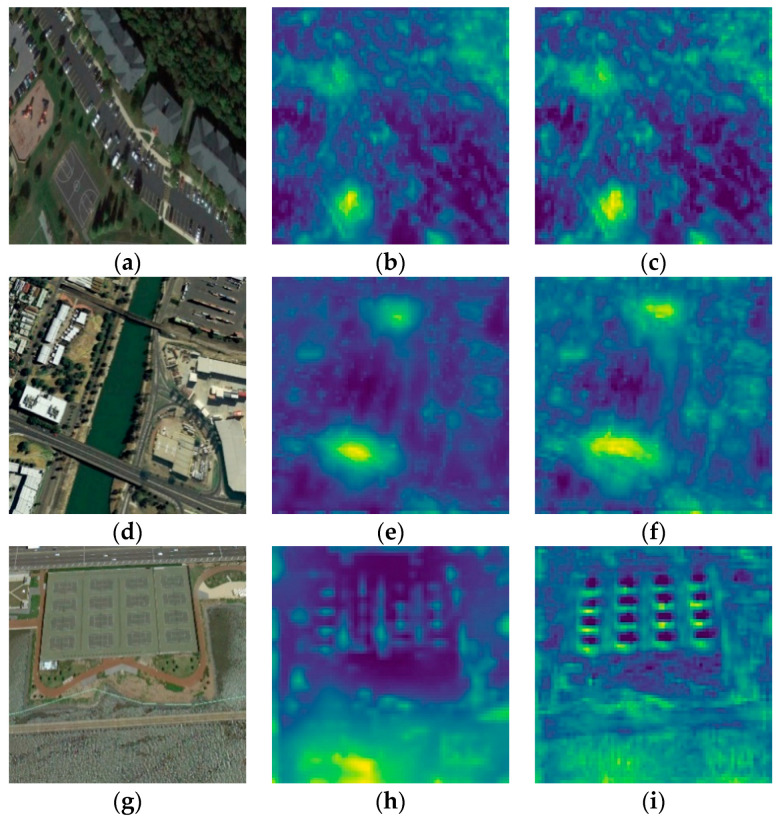
Visualization of feature maps before and after the cascade attention module. The (**a**,**d**,**g**) shows the inputs of the network. Images in (**b**,**e**,**h**) and (**c**,**f**,**i**) are feature maps before and after the cascade attention module, respectively. One channel of feature maps is shown here.

Table 4 and Table 5 show the mAP and the number of parameters in the ablation studies. Compared with results in the second row in Table 4 and Table 5, *Light Backbone* in the third row has 0.3% higher mAP (80.8% vs. 80.5%) and fewer parameters (4.3 M vs. 24.9 M, five times less than the number of parameters in the SSD). This illustrates that our lightweight backbone with lighter, wider, and deeper blocks can reduce parameters and extract rich semantic information for detection simultaneously. In the 4th row, Light Backbone + EDM shows significant improvement in the detection performance, with mAP rising from 80.8% to 86.5%, especially for small-scale categories: Ship (increasing by 12.4%), Storage tank (rising by 18.1%), Vehicle (with the highest increase by 21.9%). Therefore, by fusing low-level and high-level features, the encoding–decoding module can compensate for the insufficiency of the feature extraction for small objects in the original SSD. The number of parameters in Light Backbone + EDM increases slightly by 0.6 M compared with the module without the encoding–decoding module. In row 5, our proposed method shows improvement in AP values for low-contrast and few-texture categories, such as Tennis court (1.6% higher), Basketball court (9.3% higher), Bridge (11.7% higher). Additionally, the mAP over all categories improves by 1.7%, with only 0.8M extra parameters. To present the effectiveness of the cascade attention module, we also visualize feature maps before and after the cascade attention module in Figure 11. After the cascade attention module, Figure 11c,f,i show more obvious features in and around the targets than Figure 11b,e,h. These explain that our cascade structure with spatial and channel attention modules can guide the network to capture contextual and easily recognized features for low-contrast and few-texture targets. In the last row, our network with Leaky ReLU can also improve the mAP by 0.5%. In conclusion, compared with the original SSD with VGG16, our AF-SSD has four times fewer parameters (5.7 M vs. 24.9 M in Table 5), 8.2% higher mAP (88.7% vs. 80.5% in Table 4), and quicker detection speed (0.035 s vs. 0.042 s in Table 3). In the AF-SSD, the lightweight backbone can reduce parameters without lowering the detection accuracy. The encoding–decoding module can solve the problem of SSD having poor performance on small targets. The cascade structure with spatial and channel attention modules can perform well in detecting low-contrast and few-texture targets.

## 4. Conclusions

This paper proposes a novel one-stage framework AF-SSD with a lightweight backbone, a novel encoding–decoding module, and a cascade structure with spatial and channel attention modules. Firstly, the lightweight backbone is developed to reduce parameters, and the wider and deeper convolutional blocks in the backbone can extract features effectively and keep the high accuracy for detection. Secondly, by up-sampling and fusion, the encoding–decoding module combines low-level features with high-level features, which can enrich semantic information for small targets. Thirdly, we adopt a cascade structure with spatial and channel attention modules. We use the spatial attention module to obtain contextual information for few-texture targets. The channel attention module learns weights adaptively for each channel of a feature map, which can capture easily identifiable features for both low-contrast and few-context targets. Our AF-SSD, with mAP 88.7%, average running time 0.035 s per image, and parameters 5.7 M, outperforms most of state-of-the-art methods in the NWPU VHR-10 dataset. We carry out a series of ablation experiments to demonstrate the effectiveness of each component in the AF-SSD. Our AF-SSD with fewer parameters shows significant improvement for small, low-contrast, and few-texture targets. The experimental results verify that our AF-SSD can achieve high detection precision and fast speed at the same time.

For future works, algorithms with fast speed for geospatial object detection will be applied to embedded devices to verify their detection performance and running time during inference. Additionally, anchor-free detectors will be researched to detect multi-scale objects in remote sensing images precisely.

## Figures and Tables

**Table 1:** Our proposed lightweight backbone. Conv3x3 represents the standard convolutional layer with kernel size 3 in Figure 5a. Conv1x1 is the same as Conv3x3 except for kernel size 1. SepConv3x3 refers to depthwise separable convolution in Figure 5b. Shufflev2_block is the block in Figure 5c.

	Layer Name		Operator	Stride	Output Size
MobileNetV1	Conv1	Conv1_1	Conv3x3	2	150 × 150 × 32
		Conv1_2	SepConv3x3	1	150 × 150 × 64
	Conv2	Conv2_1	SepConv3x3	2	75 × 75 × 128
		Conv2_2	SepConv3x3	1	75 × 75 × 128
		Conv2_3	SepConv3x3	1	75 × 75 × 256
		Conv2_4	SepConv3x3	1	75 × 75 × 256
	Conv3	Conv3_1	SepConv3x3	2	38 × 38 × 512
		Conv3_2	SepConv3x3	1	38 × 38 × 512
		Conv3_3	SepConv3x3	1	38 × 38 × 512
		Conv3_4	SepConv3x3	1	38 × 38 × 512
		Conv3_5	SepConv3x3	1	38 × 38 × 512
		Conv3_6	SepConv3x3	1	38 × 38 × 512
	Conv4	Conv4_1	SepConv3x3	2	19 × 19 × 1024
		Conv4_2	SepConv3x3	1	19 × 19 × 1024
Extra Conv Layers	Conv5	Conv5	Shufflev2_block	2	10 × 10 × 512
	Conv6	Conv6	Shufflev2_block	2	5 × 5 × 256
	Conv7	Conv7_1	Conv1x1	1	5 × 5 × 128
		Conv7_2	SepConv3x3	1	3 × 3 × 256
	Conv8	Conv8_1	Conv1x1	1	3 × 3 × 128
		Conv8_2	SepConv3x3	1	1 × 1 × 256

**Table 2:** The mAP (%) of our proposed AF-SSD and state-of-the-art methods in the NWPU VHR-10 dataset. The result of SSD^#^ with VGG16 is our reproduced result with the same parameters as AF-SSD. The categories are Airplane (PL), Ship (SP), Storage tank (ST), Baseball diamond (BD), Tennis court (TC), Basketball court (BC), Ground track field (GTF), Harbor (HB), Bridge (BR), and Vehicle (VH). We bold the two highest values in each column.

Method	mAP	PL	SP	ST	BD	TC	BC	GTF	HB	BR	VH
COPD	54.6	62.3	68.9	63.7	83.3	32.1	36.3	85.3	55.3	14.8	44.0
YOLOv2	60.5	73.3	74.9	34.4	88.9	29.1	27.6	98.8	75.4	51.8	51.3
RICNN	72.6	88.4	77.3	85.3	88.1	40.8	58.5	86.7	68.6	61.5	71.1
R-P-Faster RCNN	76.5	90.4	75.0	44.4	89.9	79.7	77.6	87.7	79.1	68.2	73.2
NEOON	77.5	78.3	81.7	94.6	89.7	61.3	65.0	93.2	73.2	59.5	78.3
SSD^#^	80.5	94.3	70.9	69.6	89.7	80.0	76.2	96.9	84.7	82.3	60.2
Faster RCNN	80.9	94.6	82.3	65.3	95.5	81.9	89.7	92.4	72.4	57.5	77.8
[24]	83.8	93.4	77.1	87.5	93.0	82.7	83.8	83.7	82.5	72.5	82.3
CACMOD CNN	90.4	96.9	90.0	84.8	96.3	94.7	88.6	94.8	95.8	86.4	76.0
Ours	88.7	99.0	85.5	90.5	95.6	87.1	74.6	98.7	86.8	86.1	82.8

**Table 3:** Average running time of our proposed AF-SSD and state-of-the-art methods. The result of SSD^#^ with VGG16 is our reproduced result with the same parameters as AF-SSD. We bold the two highest values.

Method	Average Running Time (s)
COPD	1.070
YOLOv2	0.026
RICNN	8.770
R-P-Faster RCNN	0.150
NEOON	0.059
SSD^#^	0.042
Faster RCNN	0.430
[24]	0.057
CACMOD CNN	2.700
Ours	0.035

**Table 4:** The mAP (%) of ablation experiments in the NWPU VHR-10 dataset. The categories are Airplane (PL), Ship (SP), Storage tank (ST), Baseball diamond (BD), Tennis court (TC), Basketball court (BC), Ground track field (GTF), Harbor (HB), Bridge (BR), and Vehicle (VH). We bold the highest value in each column. The result of SSD^#^ with VGG16 is our reproduced result with the same parameters as AF-SSD.

Method	mAP	PL	SP	ST	BD	TC	BC	GTF	HB	BR	VH
SSD^#^	80.5	94.3	70.9	69.6	89.7	80.0	76.2	96.9	84.7	82.3	60.2
Light	80.8	90.9	73.1	72.2	92.5	82.3	72.0	98.3	81.6	85.1	59.7
Light + EDM	86.5	96.7	85.5	90.3	97.8	84.3	66.8	98.4	86.5	77.3	81.6
Light + EDM + Cascade	88.2	98.5	85.1	90.2	90.1	85.9	76.1	99.0	85.3	89.0	83.0
AF-SSD	88.7	99.0	85.5	90.5	95.6	87.1	74.6	98.7	86.8	86.1	82.8

**Table 5:** The number of parameters in the ablation studies. The result of SSD^#^ with VGG16 is our reproduced result with the same parameters as AF-SSD.

Method	Parameters
SSD^#^	24.9 M
Light	4.3 M
Light + EDM	4.9 M
Light + EDM + Cascade	5.7 M
AF-SSD	5.7 M
